# Photoacoustic imaging is a novel tool to measure finger artery structure and oxygenation in patients with SSc

**DOI:** 10.1038/s41598-022-23826-1

**Published:** 2022-11-28

**Authors:** Sarah Wilkinson, James Cummings, Sakif Zafar, Martin Kozar, Joanne Manning, Graham Dinsdale, Michael Berks, Christopher Taylor, Mark Dickinson, Ariane L. Herrick, Andrea K. Murray

**Affiliations:** 1 Division of Musculoskeletal and Dermatological Sciences, University of Manchester, Manchester, UK; 2 Department of Rheumatology, Salford Care Organisation, Northern Care Alliance NHS Foundation Trust, Manchester Academic Health Science Centre, Salford, UK; 3 Department of Physics and Astronomy, University of Manchester, Manchester, UK; 4 Centre for Imaging Sciences, Division of Informatics, Imaging and Data Sciences, University of Manchester, Manchester, UK; 5 Photon Science Institute, University of Manchester, Manchester, UK; 6 NIHR Manchester Biomedical Research Centre, University of Manchester, Manchester, UK

**Keywords:** Optics and photonics, Other photonics, Photoacoustics, Biomarkers, Prognostic markers, Health care, Medical imaging, Medical research, Translational research, Rheumatology, Connective tissue diseases

## Abstract

Systemic sclerosis (SSc)-related digital ischaemia is a major cause of morbidity, resulting from a combination of microvascular and digital artery disease. Photoacoustic imaging offers a newly available, non-invasive method of imaging digital artery structure and oxygenation. The aim of this study was to establish whether photoacoustic imaging could detect and measure vasculopathy in digital arteries, including the level of oxygenation, in patients with SSc and healthy controls. 22 patients with SSc and 32 healthy controls (HC) underwent photoacoustic imaging of the fingers. Vascular volume and oxygenation were assessed across eight fingers at the middle phalanx. In addition, oxygenation change during finger occlusion was measured at the non-dominant ring finger and the vascular network was imaged along the length of one finger for qualitative assessment. There was no statistically significant difference in vascular volume between patients with SSc and HC (mean of eight fingers; SSc, median 118.6 IQR [95.0–130.5] vs. HC 115.6 [97.8–158.9]) mm^3^. However, baseline oxygenation (mean 8 fingers) was lower in SSc vs. HC (0.373 [0.361–0.381] vs. 0.381 [0.373–0.385] arbitrary sO2 units respectively; p = 0.03). Hyperaemic oxygenation response following occlusion release was significantly lower in SSc compared to HC (0.379 [0.376–0.381] vs. 0.382 [0.377–0.385]; p = 0.03). Whilst vascular volume was similar between groups, digital artery oxygenation was decreased in patients with SSc as compared to HC, indicative of functional deficit. Photoacoustic imaging offers an exciting new method to image the vascular network in patients with SSc and the possibility to capture oxygenation as a functional measure.

## Introduction

Systemic sclerosis (SSc) is an autoimmune disease causing fibrosis of connective tissues and changes to microvascular structure and function. One of the most common morbidities, affecting over 95% of patients, is Raynaud’s phenomenon; episodic colour changes to digits due to ischaemia–reperfusion on exposure to cold and stress^1,2^. The associated cycle of digital (finger/toe) ischaemia and reperfusion, combined with structural vascular change, can lead to the development of painful, long-lasting digital ulceration that can significantly affect life quality, necessitate hospital admission and can escalate to gangrene necessitating amputation^3^. The microvascular changes that occur in SSc are well recognised and can be non-invasively imaged by techniques such as nailfold capillaroscopy; however, it is also known that in cases of severe ischaemia the digital arteries are also affected and can become occluded^4^. Thus there is likely a transition that occurs when vasculopathy progresses from microvascular to digital arterial/larger vessel disease, causing ischaemic deterioration in the tissue, predisposing to digital ulcer development. Several studies have assessed the arteries in the hands and fingers with duplex/colour/power Doppler ultrasound^5–9^. These studies indicate that arterial lumen occlusion, particularly in the fingers, is observed in patients with SSc and can be associated with the occurrence of digital ulcers. A robust method to longitudinally measure progression of large vessel vasculopathy, combined with measurement of oxygenation would increase our understanding of the underlying pathophysiological processes underpinning severe digital vasculopathy. In addition, once validated, a sensitive technique to detect and monitor early digital artery change would allow studies examining early intervention strategies and potentially prevention of escalation to tissue damage.

### Photoacoustic imaging

**Figure 1:**
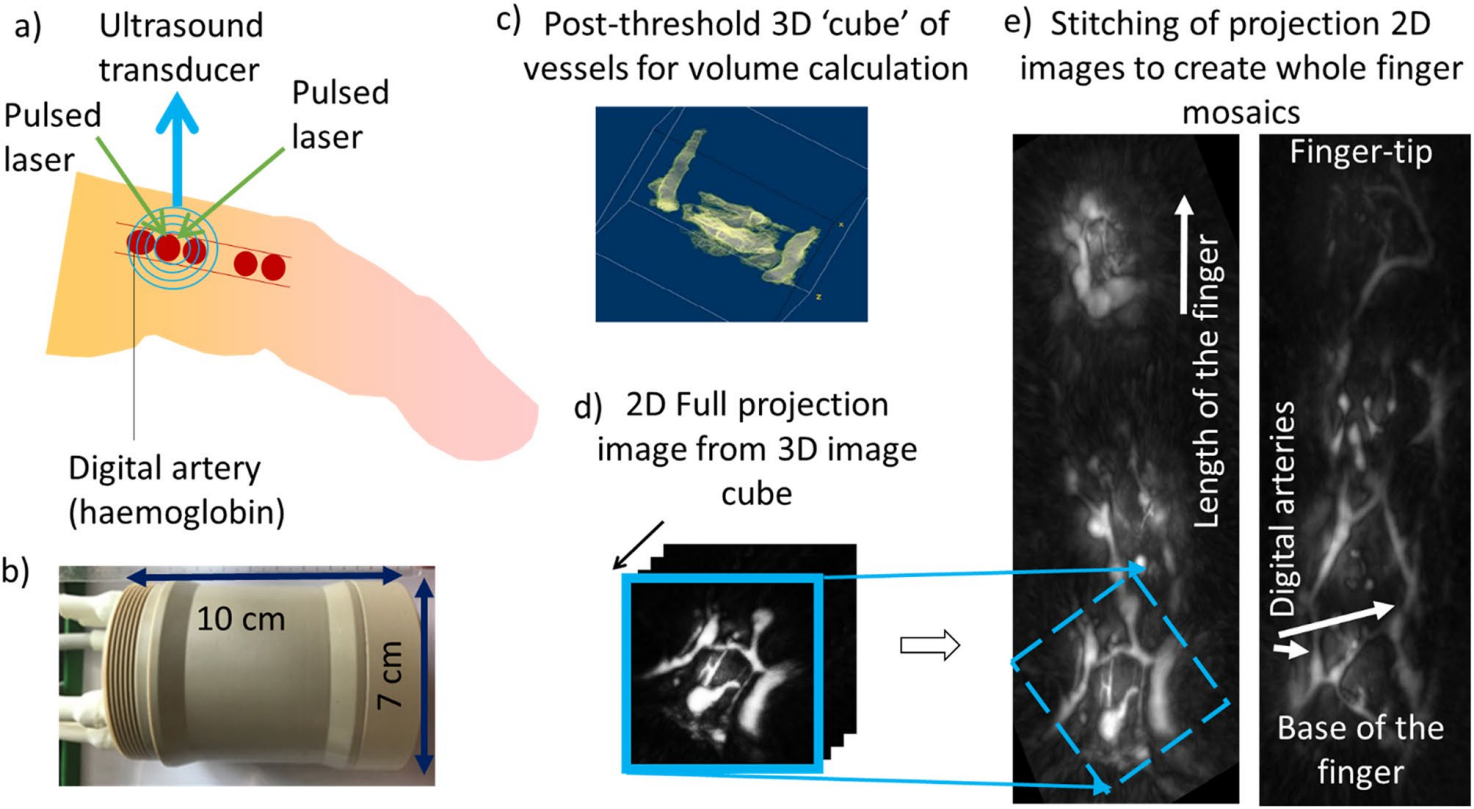
(**a**) Photoacoustic imaging technique. Pulsed laser light is incident on the tissue. The laser light is preferentially absorbed by the red blood cells due to the haemoglobin absorption spectrum. The light is converted to heat and the tissue generates acoustic waves detected by an ultrasonic transducer; (**b**) Cylindrical transducer used to collect images; (**c**) example of a post-threshold structural image generated in 3D (here the presence of haemoglobin, thus digital vessels, is indicated in lighter pixels); (**d**) representation of the creation of a projection image with frames from the cube being compressed into a 2D image. (**e**) examples of 2D projection full finger scans (left patient with SSc and right healthy control) indicating that digital arteries can be visualised in patients as well as controls facilitating quantitative measures of volume.

Photoacoustic (or optoacoustic) imaging combines the advantages of ultrasound (depth of tissue penetration) and optical imaging (absorption by specific absorbers [chromophores], e.g. haemoglobin)^10,11^. Photoacoustic imaging can be tailored to take advantage of tissue chromophores, e.g. haemoglobin, as endogenous contrast agents. The technique involves multiple wavelengths of pulsed laser light incident on the skin, with wavelengths chosen to match the absorption of oxy- and deoxy-haemoglobin. Here we use Multispectral Optoacoustic Tomography (MSOT)^12–14^. Absorbed laser light generates acoustic waves detected by an ultrasound transducer. Thus vessels containing haemoglobin in the vascular network are highlighted in images (Fig. 1); images generated can be analysed to provide both structural and functional data.

Photoacoustic imaging has been reported in a small number of studies of SSc, including oxygenation measures^15–18^. To date the structure and oxygenation along the length of the finger arteries has not been measured directly.

The overall aim of this study was to establish whether photoacoustic imaging was a suitable method to detect and measure vasculopathy in digital arteries, including the level of oxygenation, in patients with SSc and healthy controls. Our hypothesis was that vascular volume and oxygenation would be decreased in patients with SSc as compared to the healthy control group.

## Methods

### Patient and control groups

Individuals with a confirmed diagnosis of SSc (fulfilling ACR/EULAR 2013 criteria^19^) and those with no known health conditions (‘healthy control’ group) were recruited. Patients were subdivided into those with and without severe digital ischaemia; defined as current or previous digital ulceration, previous surgical debridement, amputation, or admission for intravenous vasodilatory therapy. All participants gave written informed consent and ethical approval was obtained from the National Research Ethics Service South Central, Oxford B ethics committee (17/SC/0047). All experiments were performed in accordance with relevant guidelines and regulations. Operators and patients wore appropriate protective eyewear. Patients underwent 20 min acclimatisation in a temperature controlled laboratory (23 °C) prior to imaging. Participants were asked to refrain from caffeine and nicotine for 4 h before the study.

### Equipment

A prototype MSOT technology was used for the study (Acuity, iThera Medical, Munich). The system contained a tuneable pulsed laser (650–830 nm, 30 mJ, 8 ns pulse length, 50 Hz rep rate). The 384 element 8 MHz transducer array captured signals that were band-pass filtered (100 kHz–10 MHz) and subsequently reconstructed to 2 × 2 × 1 cm image volumes through ‘Filtered back projection’ based on time of flight^20^. Image volumes had a lateral resolution of 80 µm and 50 µm in the depth dimension (with volume error therefore being 124 µm^3^).

### Imaging

The hand was placed flat, palm down, on a stable surface. Ultrasound gel was applied to the finger to be imaged (dorsal aspect) and the probe (Fig. 1b) was placed on the gel. The probe was positioned at the same point for each participant in order to capture the same region of interest. Three sets of images were obtained:

- *Imaging at the middle phalanges (8 fingers):* A single MSOT image showing the structure and ‘static’, baseline oxygenation of the vasculature was obtained from the middle phalanx, of each finger for all participants.
- *Occlusion of the non-dominant ring finger:* Each participant underwent imaging during finger occlusion of the non-dominant ring finger. A small pressure cuff was placed on the ring finger at the middle phalanx. The probe was placed on the ring finger on the dorsal aspect of the middle phalanx. Occlusion was performed by inflating the cuff with a sphygmomanometer to 200 mmHg (to ensure arterial occlusion) for two minutes. MSOT images were recoded as a series of frames (effectively a video) at baseline prior to occlusion, during occlusion, and on release.
- *Imaging of the whole finger:* A series of frames also was taken down the length of the finger (the probe was placed initially on the metacarpophalangeal joint, dorsal aspect and slowly moved to the distal end of the finger) at 800 nm, an isobestic (not dependent on the oxygenation content) wavelength providing structural measures of the vessels, independent of oxygenation.

### Image analysis

Structural image cubes (Fig. 1) taken at a wavelength of 800 nm were thresholded (truncating low intensity pixels using a histogram based cut-off) to remove noise and aid visualisation of vascular structures. This was done with a moment preserving method that involves the use of intensity based histograms^21^. Images were handled in ImageJ^22^ and bespoke analysis carried out in Java (Oracle, Tx, USA).

Oxygenation images were reconstructed from cubes taken at 700, 730, 760, 800 and 850 nm wavelengths via the technique of pseudo-inverse unmixing^23^ and thresholding to the noise level to avoid mis-classification of noisy signals. Calculation of oxygenation was from the standard equation for oxygen saturation:

$$\left[ {{\text{HbO}}_{{2}} } \right]/\left[ {{\text{Hb}} + {\text{HbO}}_{{2}} } \right],$$

where [HbO_2_] is oxy-haemoglobin and Hb is deoxy-haemoglobin; [Hb + HbO_2_] is total haemoglobin^24^.

#### Imaging at the middle phalanges

Vessel volumes (calculated as voxels above threshold multiplied by voxel volume) were calculated over the whole image cube and baseline oxygenation were calculated (as discussed above) and averaged over the eight fingers.

#### Occlusion of the non-dominant ring finger

Oxygenation data was extracted as for single image cubes (described above) at each time point.

#### Imaging of the whole finger

Each finger scan comprised a series of approximately 200 full depth projections (where 3D layers of an image are compressed into a single layer providing a 2D image from the surface of the skin down through the finger). Where possible each 2D frame had significant spatial overlap, with adjacent frames, showing the same location in part of each image, to allow overlay of these sections and thus reconstruction of finger vasculature for qualitative assessment.

### Statistical analysis

Vascular volume, baseline oxygenation and occlusion data were assessed with Mann–Whitney U tests (Stata V14, StataCorp, Texas, USA) to assess differences between the patients with SSc and the healthy controls. Data were compared at a finger level and as a mean of all 8 fingers. Patients with SSc were subdivided into those with a history (SSc_isch_) vs. those with no history (SSc_no_isch_) of severe digital ischaemia in order to assess whether any findings would be more marked in the severe ischaemia group. Due to the small numbers, no formal statistics were carried out on these patient subgroups of history vs. no history of severe digital ischaemia.

## Results

Twenty two patients with SSc (including 6 with severe digital ischaemia) and 32 healthy controls took part in the study (demographics and characteristics in Table 1.

**Table 1:** Demographic and clinical characteristics of participants.

Demographics	Healthy control group N = 32	SSc group N = 22
Age in years, median (IQR)	49 (40–58)	63 (52–67)
Female, N(%)	23 (72)	18 (82)
Smoker, N(%)	1 (3)	3 (14)
**SSc disease subtype**		
Limited cutaneous N(%)		17(77)
Diffuse cutaneous N(%)	N/A	5 (22)
Duration of Raynaud’s phenomenon in years, median (IQR)	N/A	19 (6–31)
Duration of SSc from first non-Raynaud’s manifestation in years, median (IQR)	N/A	13.5 (5–26)
Current ulcers, yes N(%)	N/A	2 (9)
Severe digital ischaemia (defined by history of inpatient intravenous vasodilator therapy, debridement or amputation or previous ulcers) N(%)	N/A	6 (27)

N = number; IQR = interquartile range.

### Imaging at the middle phalanges

**Figure 2:**
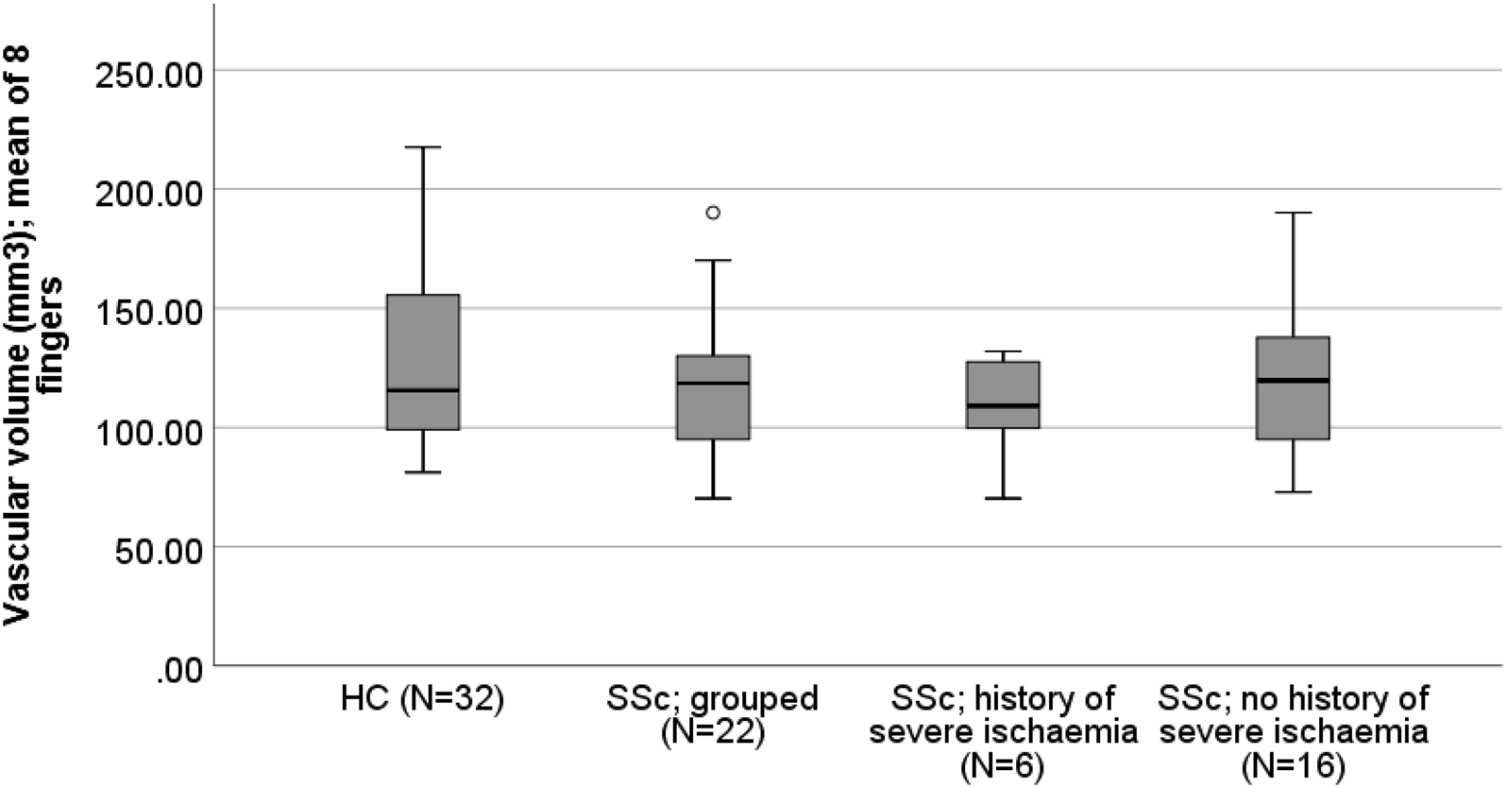
Boxplot of mean vascular volume (mm^3^) over all eight fingers’ middle phalanges. Comparison between healthy controls and patients with SSc and also the two subsets of SSc; those with a history of severe ischaemia and those without a history.

Mean vascular volume of the eight fingers is shown in Table 2 and Fig. 2 for patients with SSc and controls and for the subsets of patients with (SSc_isch_, N = 6) and without (SSc_no_isch,_ N = 16) severe ischaemia. There was no statistically significant difference in vascular volume between the SSc and healthy control groups (median 118.6 (95.0–130.5) vs. 115.6 (IQR 97.8–158.9) mm^3^; p = 0.89. When patients with SSc were compared based upon history of severe digital ischaemia, patients with a history of ischaemia had a vascular volume of 109.1 [IQR 92.3–128.7] vs. patients without a history 118.6 [95.0–130.1] mm^3^.

**Table 2:** Vascular volume (mm^3^). Data for healthy controls and patients with SSc and for the patient subsets subdivided into those with and without a history of severe digital ischaemia.

	Comparison of full data set			Comparison of SSc ischaemia	
	Healthy control median (IQR)	SSc median (IQR)	P-Value (HC vs SSc)	SSc history of ischaemia median (IQR)	SSc no history of ischaemia median (IQR)
	N = 32	N = 22		N = 6	N = 16
Vascular volume (mm^3^); mean of 8 fingers	115.6 (97.8–158.9)	118.6 (95.0–130.5)	0.89	109.1 (92.3–128.7)	118.6 (95.0–130.1)

N = number; IQR = interquartile range; *p < 0.05 is significant.

**Figure 3:**
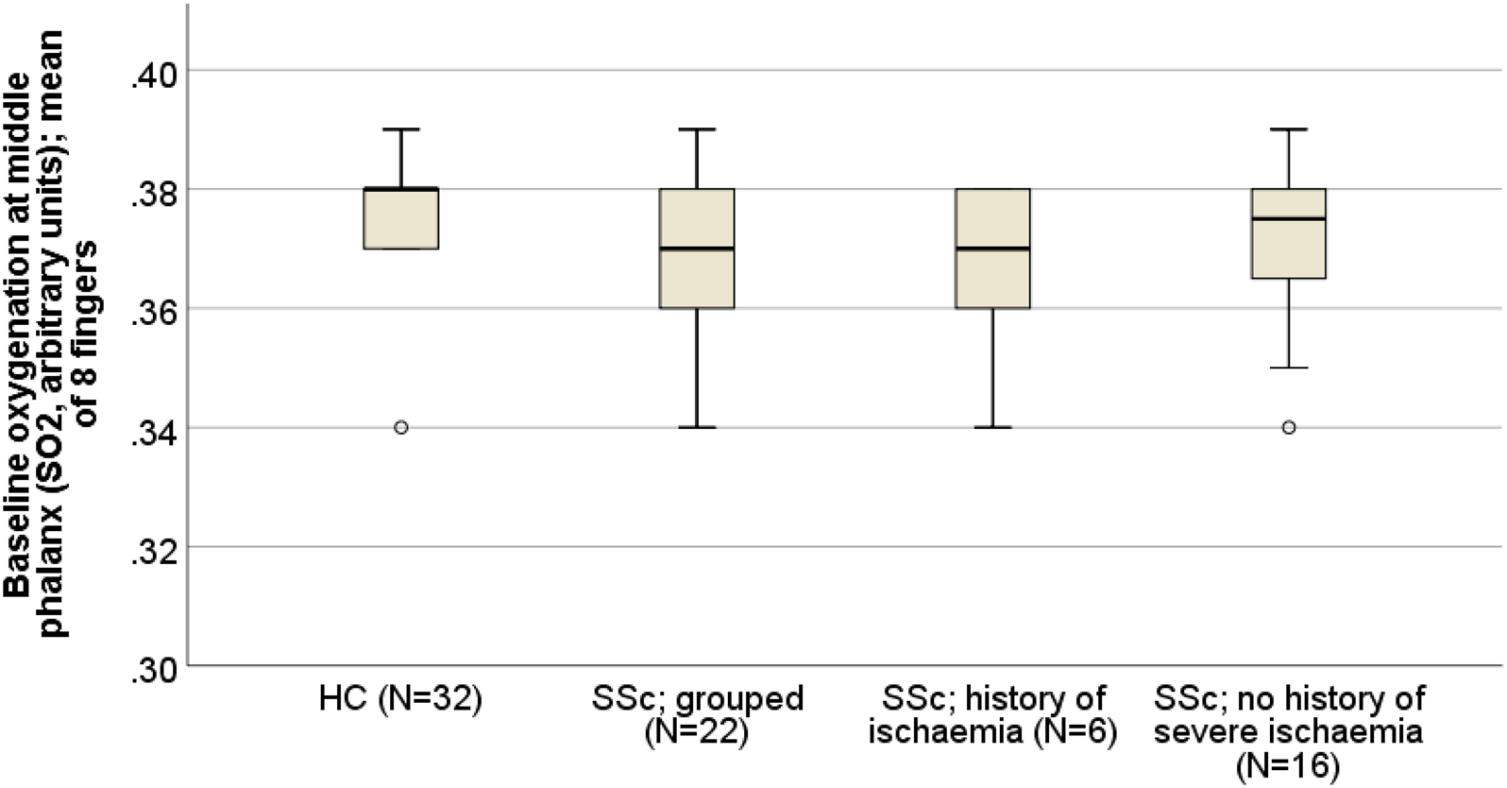
Boxplot of mean baseline oxygenation (sO2, arb units) over all eight fingers’ middle phalanges. Comparison between healthy controls and patients with SSc and also the two subsets of SSc; those with a history of severe ischaemia and those without a history.

Baseline oxygenation is shown in Table 3 and Fig. 3. Oxygenation was decreased in the SSc vs control group (0.373 [0.361–0.381] vs. 0.381 [0.373–0.385] arb units [AU]; p = 0.03). Oxygenation values for the group with a history of digital ischaemia were 0.370 [0.356–0.379] vs. 0.374 [0.363–0.382] AU for patients with SSc and no history of ischaemia.

**Table 3:** Oxygenation (arbitrary units) mean 8 fingers (middle phalanges). Data for healthy controls and patients with SSc and for the patient subsets subdivided into those with and without a history of severe digital ischaemia.

	Comparison of full data set			Comparison of SSc ischaemia	
	Healthy control median (IQR)	SSc median (IQR)	P-Value (HC vs SSc)	SSc history of ischaemia median (IQR)	SSc no history of ischaemia median (IQR)
	N = 32	N = 22		N = 6	N = 16
Baseline oxygenation at middle phalanx (sO2, arbitrary units); mean of 8 fingers	0.381 (0.373–0.385)	0.373 (0.361–0.381)	0.03*	0.370 (0.356- 0.379)	0.374 (0.363–0.382)

N = number; IQR = interquartile range; *p < 0.05 is significant; sO2 = saturated oxygenation.

### Occlusion of the non-dominant ring finger

**Figure 4:**
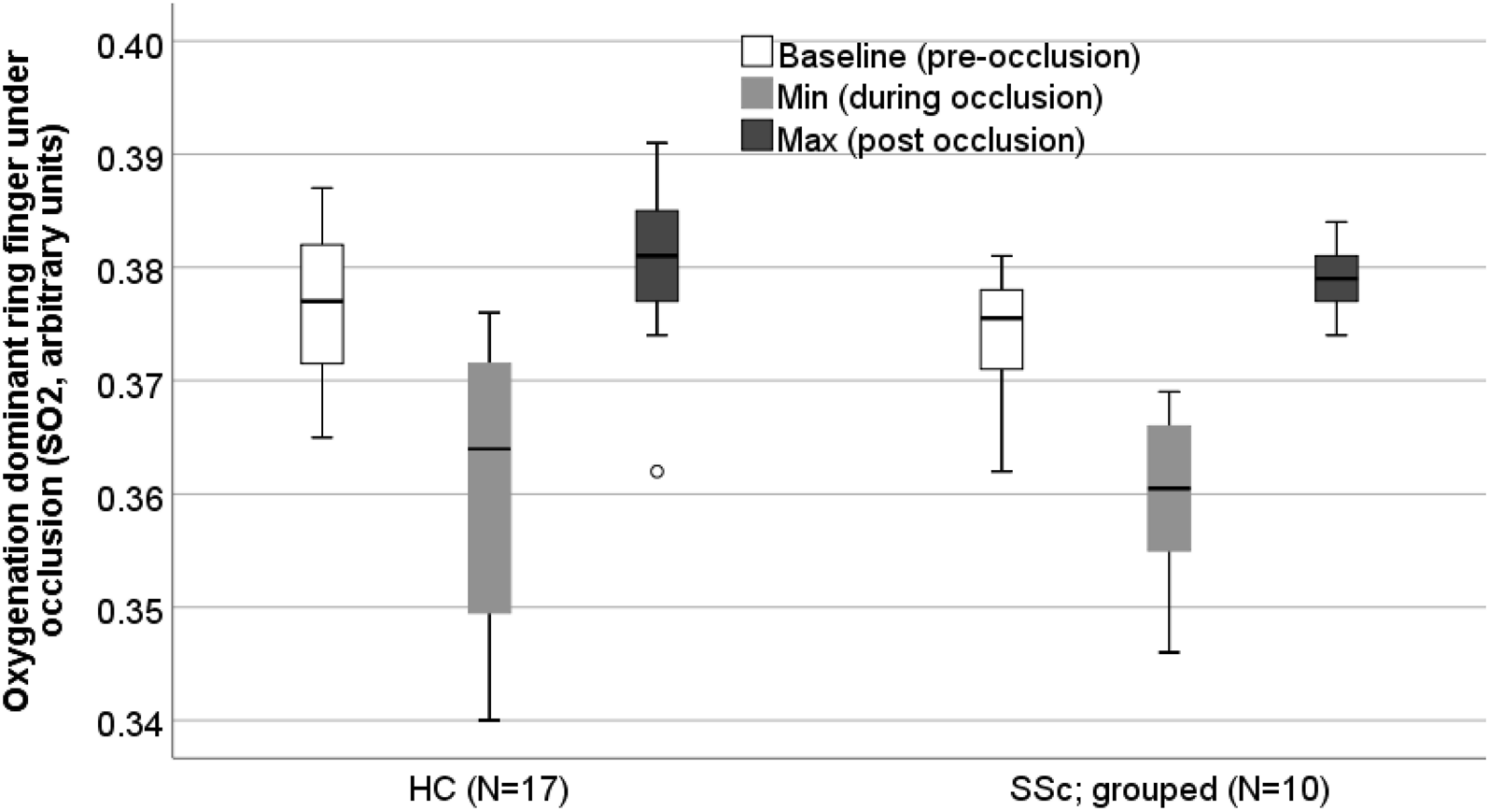
Boxplot of oxygenation (sO2, arb units) before, during and after occlusion of the non-dominant ring finger. Comparison between healthy controls and patients with SSc at each time point.

Oxygenation change during occlusion is shown in Table 4 and Fig. 4. Due to noise, movement artefacts and processing issues on the occlusion videos, data could be obtained from only 17 controls and 10 patients with SSc. Maximum oxygenation value following occlusion release was significantly lower in the patient vs control group (0.379 [0.376–0.381] vs. 0.382 [0.377–0.385] AU; p = 0.03). For patients with a history of ischaemia (N = 3) the baseline and maximum value after occlusion were lower compared to those with no history (N = 7).

**Table 4:** Oxygenation at baseline, during finger occlusion (minimum value) and hyperaemia upon release from occlusion (maximum value). Data for healthy controls and patients with SSc and for the patient subsets subdivided into those with and without a history of severe digital ischaemia.

	Comparison of full data set			Comparison of SSc ischaemia	
	Healthy control median (IQR)	SSc median (IQR)	P-Value (HC vs SSc)	SSc history of ischaemia median (IQR)	SSc no history of ischaemia median (IQR)
**Oxygenation dominant ring finger under occlusion (sO2, arbitrary units)**					
	N = 17	N = 10		N = 3	N = 7
Baseline oxygenation	0.377 (0.371–0.382)	0.376 (0.371–0.378)	0.63	0.371 (0.362)	0.377 (0.373–0.379)
Minimum oxygenation (during occlusion)	0.364 (0.350–0.372)	0.361 (0.354–0.367)	0.81	0.355 (0.350)	0.361 (0.357–0.368)
Maximum oxygenation (upon release of occlusion)	0.382 (0.377–0.385)	0.379 (0.376–0.381)	0.03*	0.377 (0.374)	0.379 (0 378–0.382)

N = number; IQR = interquartile range; *p < 0.05 is significant; sO2 = saturated oxygenation.

### Imaging of the whole finger

**Figure 5:**
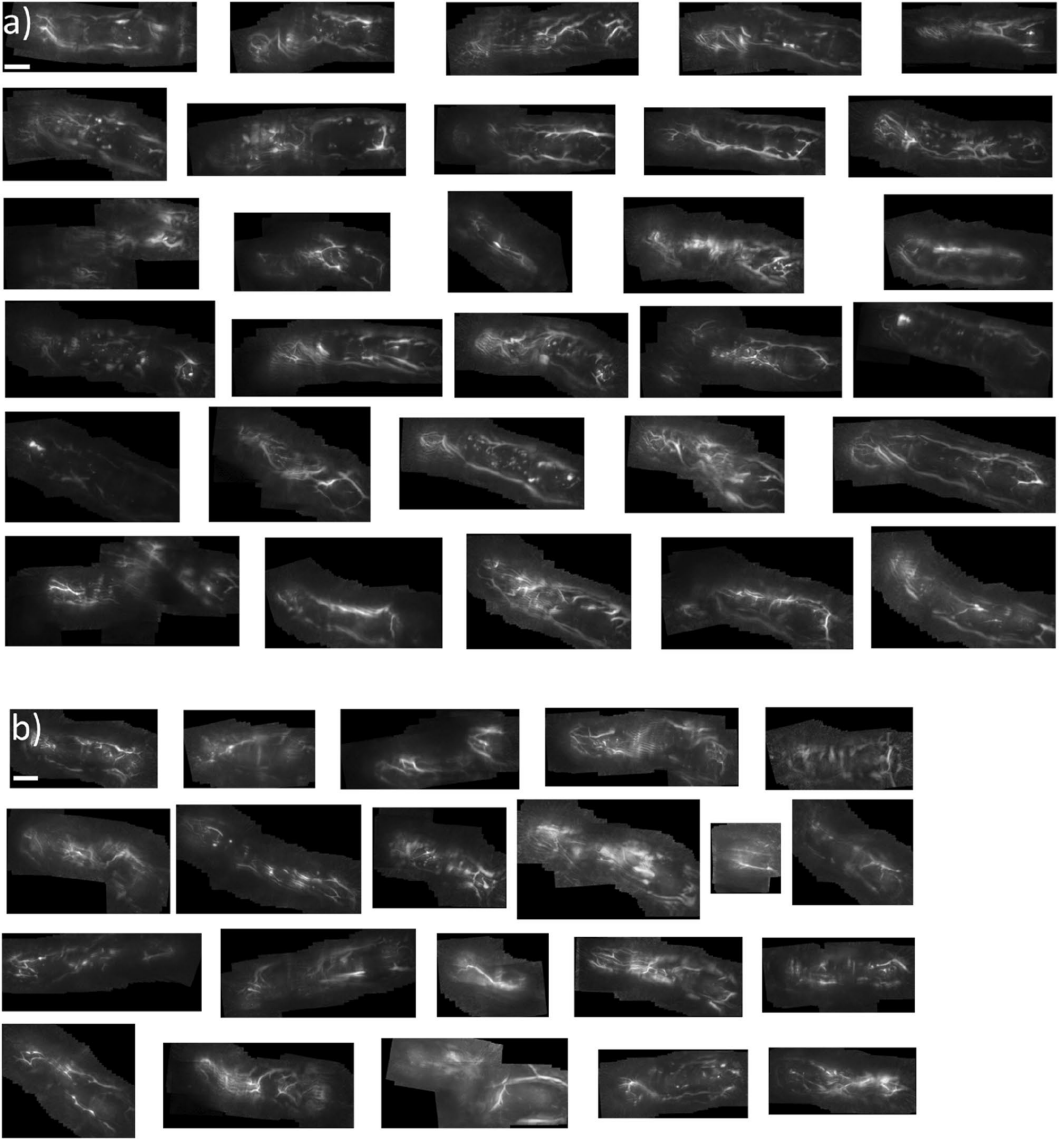
Reconstruction of finger length digital arterial images; (**a**) healthy controls (N = 30 reconstructed) and (**b**) patients with SSc (N = 21 reconstructed). Note that in some images overlap of frames leading to duplicate ‘ghosting’ of the vessels making vascular volume measures difficult. Scale bar in the first image of each set represents approximately 1 cm length.

Due to noise and movement artefacts fully automated reconstruction of the images from the videos could not easily be carried out; no further data extraction was performed. Examples of reconstructed images of a control and a patient with SSc, are shown in Fig. 1d and Fig. 5.

## Discussion

This study included larger numbers of patients with SSc and controls than have been previously reported with photoacoustic imaging, and included examination of the digital arteries, which have not been studied in this way previously with photoacoustic imaging. We have shown that in patients with SSc, both digital artery volume and oxygenation can be measured non-invasively. This is a very exciting finding, potentially providing a new methodology to investigate pathophysiology of digital artery disease in patients with SSc, and to monitor progression and treatment response. It is also very possible that photoacoustic imaging could complement nailfold capillaroscopy^25^ in helping to identify those patients at highest risk of digital ulceration: patients with the most severe microvascular and digital artery disease are those most likely to progress to tissue injury.

Vascular volume was similar between the healthy control and patient groups; however, baseline oxygenation (mean 8 fingers) was lower in patients with SSc as compared to controls, as was the hyperaemic value of oxygenation, post-occlusion. This indicates a functional deficit at the digital artery level and a possible precursor to structural change and a contributor to tissue injury. That hyperaemic but not occluded oxygenation differed between the groups suggests that once occluded for 2 min both the healthy control and patient cohort have similar oxygenation depletion but as the occlusion is released in the patient cohort the vessels, which may be fibrosed due to disease, are less elastic and thus both blood flow and oxygenation take longer to return to baseline values.

Previous studies of photoacoustic imaging in SSc have been in smaller cohorts. Initial photoacoustic, structural, imaging of in vivo healthy control finger blood vessels has been reported in several studies using various transducer geometry set-ups including Sun et al.^26^ and van Es et al.^27^. More recent studies have included oxygenation measures^28–30^. These studies include Ahn et al.^28^ who imaged a single healthy control’s nailfold capillaries under brachial occlusion with photoacoustic microscopy. Huang et al.^29^ and Deng et al.^30^ separately developed their own photoacoustic computed tomography systems and measured oxygenation at baseline (with no dynamic stimulus) through larger vessels observed in finger cross-sections (axial plane) in four and one healthy control(s) respectively.

There are a limited number of studies investigating the vasculopathy of patients with SSc^15–18^; some studying the microvasculature rather than larger vessels. Both Eisenbrey et al.^17^ and Daoudi et al.^18^ focused on high resolution imaging in the fingertip observing and measuring capillaries in the nailbed where microvascular abnormalities are known to occur. Eisenbrey et al.^17^ measured nailbed oxygenation in patients with SSc (N = 7) and controls (N = 15) before and after cold challenge (15 °C for 1 min). A larger decrease in oxygenation level in the SSc group following cold challenge could be used to discriminate between patients with SSc and controls (area under the ROC curve = 0.91)^17^. Daoudi et al.^18^ imaged, again at the nailbed, 17 patients with SSc, 5 primary Raynaud’s phenomenon and 9 healthy controls. Both studies identified differences between patients with SSc and those without microvascular involvement. In addition, two studies by Aguirre et al.^31^ and Nitkunanantharajah et al.^32^ imaged nailfold capillaries but did not measure oxygenation.

Looking at larger vessels within the finger, Liu et al.^15^ carried out a pilot study investigating multispectral photoacoustic elastic tomography, a variation of photoacoustic imaging, to image skin thickness and haemoglobin in a cross-section of the middle phalanx. They observed increased stiffness, decreased oxygenation and haemoglobin levels in the fingers of the patient group (three very severely affected patients). Masthoff et al. identified decreased oxygenated haemoglobin in the fingers of patients with SSc (N = 7) as compared to controls (N = 8) at baseline (with no dynamic challenge)^16^.

Thus our study is novel in several ways. Our study has a larger number of patients with SSc and controls as compared to previous studies over a range of severities (although we acknowledge still a small number due to the nature of a rare disease cohort). Of the two previous studies looking at larger vessels in the finger, we have studied images taken along the finger (in the sagittal plane, rather than cross-sectionally as in the paper by Liu et al.^15^. This provides complementary data to Liu et al., allowing visualisation along the length of the vessels. The study by Masthoff et al.^16^ imaged the finger in a similar way to our study. The authors measured haemoglobin (oxygenated, deoxygenated and total); in our study we have measured vascular volume and sO2 levels. In addition we have carried out a finger occlusion as a dynamic challenge to assess the change in vessel function with time.

The digital arteries can also be examined with (Doppler/power) ultrasound. Several studies have applied ultrasound techniques to image individual artery structure and perfusion in the hands of patients with SSc, and in some instances finding an association between arterial disease severity and digital ulceration^5–9^. Photoacoustic imaging is complementary to ultrasound providing the possibility of combining three-dimensional imaging with measurement of oxygenation.

Although our study was larger than other photoacoustic imaging studies, one limitation was the small subset of patients with severe digital ischaemia. A larger study including patients with and without a history of severe digital ischaemia would enable better understanding of the contribution of digital artery disease to chronic ischaemia in patients with SSc and related conditions. Another limitation of the oxygenation measures is that the prototype system was uncalibrated; with Hb and HbO2 measures being in arbitrary units. Going forwards a calibration would be beneficial to allow better comparison of future data to other studies. Prospective studies, following patients with SSc over time, will help to inform whether using photoacoustic imaging might be a future outcome measure in early-phase proof of concept clinical trials. In conclusion, this study has demonstrated the potential of photoacoustic imaging to provide both structural and functional measurements of SSc-related digital vascular disease: this should be exploited to increase our understanding of SSc vasculopathy including its prevention and treatment.

## Data Availability

Data is available from the authors. Please contact andrea.murray@manchester.ac.uk or sarah.wilkinson@manchester.ac.uk.
